# Cost-effectiveness analysis of escalating to natalizumab or switching among immunomodulators in relapsing-remitting multiple sclerosis in Italy

**DOI:** 10.1186/s12913-019-4264-1

**Published:** 2019-06-28

**Authors:** Gianluca Furneri, Laura Santoni, Chiara Ricella, Luca Prosperini

**Affiliations:** 1 EBMA Consulting, Melegnano (Milan), Italy; 2 Biogen Italy, Milan, Italy; 3 Department of Neurosciences, S. Camillo-Forlanini Hospital, Rome, Italy

**Keywords:** Multiple sclerosis, Natalizumab, Glatiramer acetate, Interferon-beta, Cost-effectiveness, Markov

## Abstract

**Background:**

Published literature suggests that early treatment with natalizumab (“escalation strategy”) is more effective than switch within the same class of immunomodulators (interferons/glatiramer acetate, “switching strategy”) in relapsing-remitting multiple sclerosis (RRMS) patients who failed first-line self-injectable disease-modifying treatment (DMT). The present analysis aims to evaluate the cost-effectiveness profile of escalation strategy vs. switching strategy, adopting the Italian societal perspective.

**Methods:**

A lifetime horizon Markov model was developed to compare early escalation to natalizumab vs. switching among immunomodulators, followed by subsequent escalation to natalizumab. The two compared treatment algorithms were: a) early escalation until progression to Expanded Disability Status Scale (EDSS) = 7.0 vs. b) switching until EDSS = 4.0, followed by escalation until EDSS = 7.0. The model analyzed social costs, quality-adjusted survival and effects of therapies in prolonging time without disability progression and burden of relapses. Clinical data were mainly extracted from a published observational study.

**Results:**

Lifetime costs of early escalation to natalizumab and switching among immunomodulators amounted to €699,700 and €718,600 per patient, respectively. Early escalation was associated with prolonged quality-adjusted survival (11.19 vs. 9.67 QALYs, + 15.8%). A slight overall survival increase was also observed (20.10 vs. 19.67 life years). Both deterministic and probabilistic sensitivity analyses confirmed the robustness of findings.

**Conclusions:**

Adopting the Italian social perspective, early escalation to natalizumab is dominant vs. switching among immunomodulators, in RRMS patients who do not respond adequately to conventional immunomodulators.

**Electronic supplementary material:**

The online version of this article (10.1186/s12913-019-4264-1) contains supplementary material, which is available to authorized users.

## Background

Multiple Sclerosis (MS) is a chronic condition, affecting young adults in the active working phase, with a significant economic and social burden [1, 2]. In most cases, patients with relapsing-remitting MS (RRMS) suffer from episodes of neurological deterioration (relapses), between periods of complete or partial remission. Transition to secondary progressive multiple sclerosis (SPMS), with or without superimposed relapses, can occur after an initial relapsing-remitting (RR) phase, leading to accumulation of irreversible disability [3]. Optimal treatment strategy then aims to minimize the occurrence of relapses and prolong the time to disability progression. As first-line treatment, RRMS patients can receive self-injectable disease-modifying treatments (DMTs), namely interferon beta (IFN) or glatiramer acetate (GA). However, a significant proportion of patients experience disease activity despite first-line DMT treatment [4]. At this stage, neurologists can decide whether switching treatment to another immunomodulator (i.e. from GA to IFN, or vice versa, or from a lower to a higher dose and/or more frequently administered IFN, hereafter called “switching strategy”) or initiating treatment with second-line therapy (i.e. from GA or IFN, to a high-efficacy DMT such as natalizumab, hereafter called “escalation strategy”) [5]. Several studies have shown that escalating to second-line therapy is more effective than switching [6, 7]. Although this evidence supports escalation from a clinical perspective, prescription of second-line treatment could lead to a relevant increase of therapeutic costs, being second-line options more expensive than first-line immunomodulators. Therefore, the economic investment for escalation should be measured against the incremental clinical benefit over switching. This analysis aims to evaluate cost-effectiveness of early escalation to natalizumab vs. switching among immunomodulators, followed by late escalation to natalizumab, in patients affected by RRMS who have failed first-line treatment with either IFNs or GA [6], adopting the Italian Societal perspective.

## Method

### Design and parameters

The present analysis is an economic elaboration of a previously published clinical study, conducted by Prosperini et al. [6]. Patients enrolled in this study had > 2 relapses, or 1 relapse associated with sustained disability worsening while receiving first-line DMT for at least 1 year, according to the past Italian Medicine Agency rules for escalation to natalizumab [8, 9]. In the study, patients who failed first-line therapy with one of the available IFNs or GA were split into two groups: patients switching among different IFN formulations, or from IFN to GA, or vice versa (switching strategy, SWI); patients receiving natalizumab (escalation strategy, ESC). Duration of follow-up was 24 months. At the end of the study, a larger proportion of patients were free from relapse (*p* < 0.0001), disability progression (*p* = 0.0045), magnetic resonance (MRI) activity (*p* = 0.0003), and combined activity (p < 0.0001), in the ESC group than in the SWI group.

**Fig. 1:**
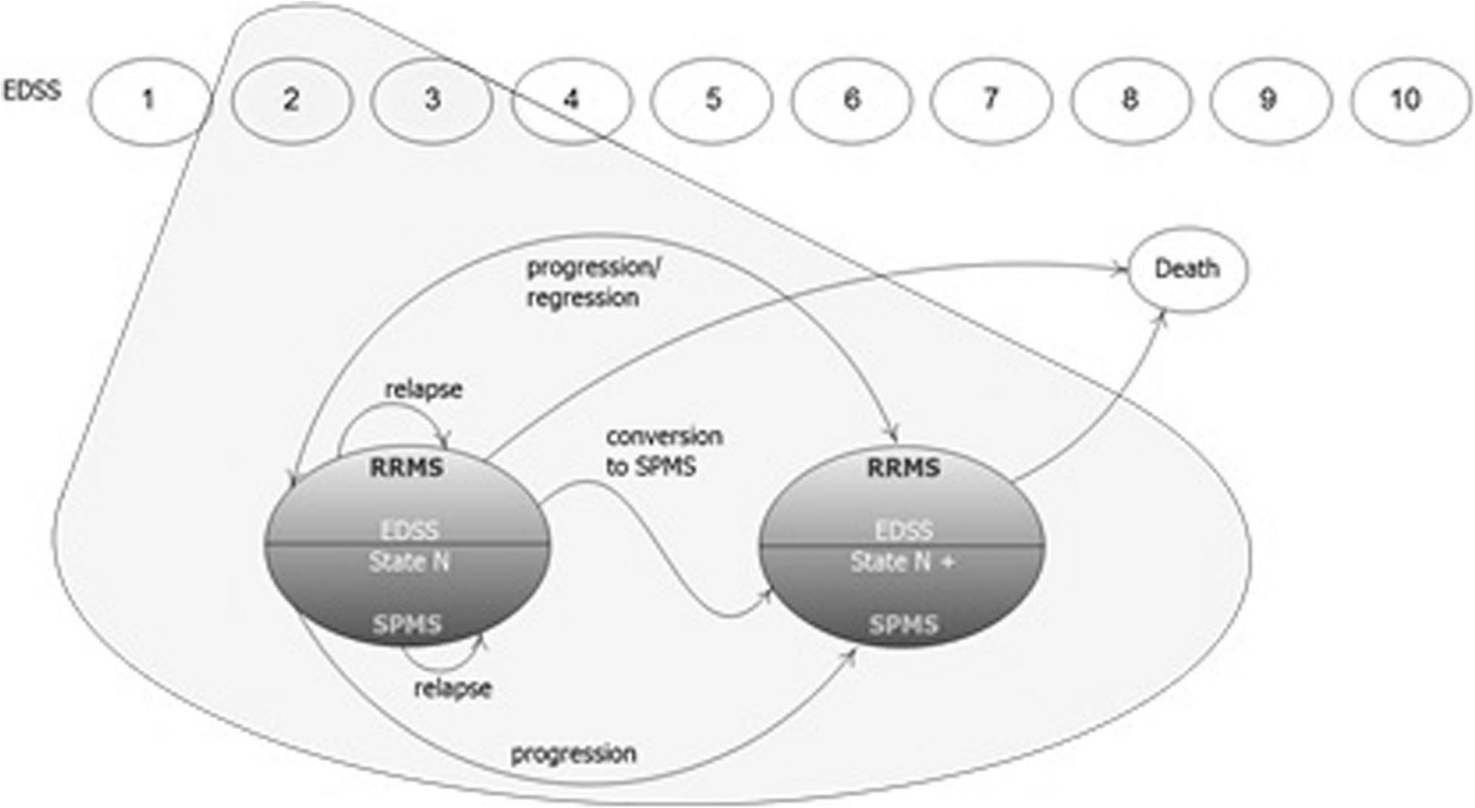
Scheme of the Markov model [15]

The findings of this study were used to develop a Markov model (Fig. 1) projecting the clinical and economic outcomes of ESC vs. SWI over a lifetime horizon (50 years) and comparing two treatment strategies: i) early escalation (ESC), in which patients were treated with natalizumab until Expanded Disability Status Scale (EDSS) = 7.0 was reached [10]; ii) switching (SWI), in which patients received IFN/GA until EDSS = 4.0, and then were switched to natalizumab (late escalation) and treated until EDSS = 7.0 was reached. No efficacy waning effects were modelled for natalizumab (over time), consistently with the findings of a large real-world, observational, prospective study of patients with RRMS, showing that the risk of disability progression in natalizumab-treated patients was relatively low in the long-term [11, 12]. Patients in both groups did not receive further disease-modifying treatment after EDSS> 7.0 [13–15]. The model was developed to measure three main clinical outcomes: disability progression, incidence of relapses and mortality.

In the model, pharmacological treatment of RRMS (with either ESC or SWI) reduces both disability progression and incidence of relapses, and improves quality of life, with an indirect impact on life expectancy [16–20]. Over time, EDSS score of each patient could increase (disability progression), decrease (disability regression), or remain stable (disability maintenance). Additionally, patients had a certain probability of progressing to SPMS. It was assumed that: i) treatments did not have any effect in preventing transition from RRMS to SPMS and in delaying disability progression while in the SPMS form; ii) patients affected by SPMS did not receive any DMT.

The model simulates mortality, disability progression (measured through EDSS), relapse occurrence and transition to secondary progressive multiple sclerosis (SPMS). As a consequence, the model provides estimates of overall survival (Life Years, LYs), quality-adjusted survival e (Quality-Adjusted Life Years, QALYs), overall costs, and finally of the incremental cost effectiveness ratio (ICER) per QALY gained.

The Italian Societal perspective was adopted. Outcomes and costs were evaluated over a 50-year time horizon (lifetime horizon) and discounted at 3.50%, in line with technology appraisal best practices, recommended by National Institute for Health and Care Excellence (NICE) [21]. A complete list of the input data used in this analysis is shown in Additional file 1: Table S1-S6.

### Clinical data

The cohort of RRMS patients enrolled in the study conducted by Prosperini et al. [6] was used to run the present analysis. The mean age of the cohort was 35.3 years (SD: 8.3 years); 65.8% of patients were females. The mean number of relapses during the year preceding enrolment was 1.7 (SD: 0.7), and the mean patient EDSS score was 2.6 (SD: 1.1). Most patients (85.50%) had EDSS< 4 at the time of enrolment. Patients in the SWI group were mainly treated with high dose beta IFN (IFN beta 1a 44 mcg subcutaneous, 58.4%, or IFN beta 1b, 8.7%) or with GA (32.9%), reflecting quite well the Italian practice on first-line treatment of RRMS.

Data on the incidence of relapses were extracted from a mixed treatment comparison (MTC) used in previous Italian economic evaluations [22–24], and then combined with results of Prosperini et al. [6]. In absence of treatment, patients affected by RRMS and SPMS had an annualized relapse rate (ARR) depending on the EDSS score (Table 1). The ARRs for the RRMS form for EDSS scores 0–7 were retrieved from Prosperini et al. [6]. The remaining relapse rate data (RRMS form, EDSS scores 8–10; SPMS, all EDSS scores) were retrieved from multiple sources [25–27], due to a lack of data in the Prosperini study [6].

**Table 1:** Relapse rate, in absence of treatment

	EDSS 1	EDSS 2	EDSS 3	EDSS 4	EDSS 5	EDSS6	EDSS 7	EDSS 8–10
Relapse rate prior to switch/escalation, RRMS (# events/year)	1.7534 [6]	1.7534 [6]	1.6698 [6]	1.7966 [6]	1.3793 [6]	1.5556 [6]	1.5556 [6]	0.1555 [25–27]
Relapse rate, SPMS (# events/year)	10.0000 [25–27]	0.3147 [25–27]	0.6020 [25–27]	0.5146 [25–27]	0.1604 [25–27]	0.1387 [25–27]	0.1041 [25–27]	0.1041 [25–27]
Patient distribution at model baseline (%)	25.61% [6]	37.19% [6]	20.70% [6]	10.18% [6]	6.32% [6]	0.00% [6]	0.00% [6]	0.00% [6]

*EDSS* expanded disability status scale, *RRMS* relapsing-remitting multiple sclerosis, *SPMS* secondary progressive multiple sclerosis

According to the MTC, immunomodulators reduced the risk of ARR of 33–36% in the RRMS cohort [22]. This effect was consistent with the findings observed in the Prosperini study, where the weighted treatment reduction effect observed in the switching group vs. baseline was 0.6574 (− 34%; average treatment effect weighted by treatment distribution [6]). The resulting effect associated with natalizumab on ARR was 0.3024, as escalation to natalizumab reduced the relapse risk by 54% vs. switching [6] (RR: 0.46; IC 95%: 0.31–0.68).

Annual transition probabilities for disability progression (measured through EDSS score) were calculated using patient-level data on EDSS change from enrolment to Month 24 [6]. The annual probability was calculated as the average of the EDSS change between Month 12 vs. baseline, and Month 24 vs. Month 12. These probabilities were used to set transition probabilities in the RRMS state. In the ESC group, probabilities of − 1 point, 0 point, + 1 point, + 2 points, + 3 points of EDSS change vs. baseline were: 0.0236, 0.8727, 0.0849, 0.0189, 0.000, respectively. In the SWI group, probabilities of − 1 point, 0 point, + 1 point, + 2 points, + 3 points of EDSS change vs. baseline were: 0.0000, 0.8199, 0.1553, 0.0217, 0.0031 respectively. The Markov model was finalized using two additional clinical inputs: i) probabilities of transition from the RRMS form to the SPMS form; ii) probabilities of disability progression for SPMS patients. Both sets of data were retrieved from the London Ontario dataset, one of the longest and most complete observational registries on multiple sclerosis, collecting MS data from 1972 and 2000 [28–30].

The annual incidence of adverse events (AEs) by treatment was retrieved from a systematic literature review [22, 24, 31]. AEs were also classified as serious and non-serious, to consider their different effect on costs and outcomes. Mortality rates, stratified by age and gender, were retrieved from national registries of the Italian Statistics Institute (ISTAT) [32]. Mortality rates of the general population were adjusted by the additional death risk attributable to multiple sclerosis, depending on both MS form and EDSS level [33].

### Quality of life data

Utilities of patients affected by RRMS, stratified by EDSS level, were retrieved from recent pivotal clinical trials [34, 35] measuring quality of life with EuroQoL EQ5D assessments for each EDSS state. Utilities associated with the SPMS form and relapse-related disutilities were determined adjusting the above-mentioned utilities for the RRMS form by negative coefficients retrieved from a survey conducted in the UK, evaluating the QoL deterioration due to disease progression [36]. These disutility factors were − 0.0092 and − 0.0437, for transition to SPMS and relapse occurrence, respectively.

Disutilities associated with treatment-related AEs and relapses were also incorporated in the model. AE-related disutilities depended on type and grade (serious vs. non-serious) of the adverse events and had a temporary, reversible effect on patients’ quality of life (duration range: 1 day - 6 months per year). These values were estimated and validated through clinical expert opinion.

### Economic data

This economic analysis was conducted adopting the Italian Societal perspective and considering the cost of disability, treatment acquisition, administration, monitoring, relapses, AEs, productivity loss and non-healthcare direct costs. Disability-related costs were retrieved from the study conducted by Karampampa et al. [1]. Table 2 reports disability-related costs included in the model, expressed in Euro, November 2015 [37].

**Table 2:** Disability-related costs (direct and indirect). Elaborated from [1]

Type of disease/type of cost	EDSS 0 (€)	EDSS 1 (€)	EDSS 2 (€)	EDSS 3 (€)	EDSS 4 (€)	EDSS 5 (€)	EDSS 6 (€)	EDSS 7 (€)	EDSS 8–10 (€)
RRMS direct	201	201	201	636	636	636	636	5708	5708
RRMS indirect	1143	1143	1143	11,847	11,847	11,847	11,847	28,411	28,411
SPMS direct	5331	5331	5331	18,894	18,894	18,894	18,894	9589	9589
SPMS indirect	4096	4096	4096	31,559	31,559	31,559	31,559	64,948	64,948

RRMS 0–6: only costs for co-medications. Not included the costs for DMTs and other disease management costs (e.g., administration, monitoring, etc.) considered in other calculation sections of the model. EDSS: expanded disability status scale. RRMS: relapsing-remitting multiple sclerosis. SPMS: secondary progressive multiple sclerosis. Direct costs include only the healthcare direct costs. The non-healthcare direct costs were included among the indirect costs

The economic impact of pharmacological treatment was expressed as net annual cost per patient, using ex-manufacturer price of single drug packs, and subtracting all rebates applied to the Italian National Healthcare System (NHS, Table 3). Administration costs were assumed equal to €0 for all the treatments included in the analysis (as IFNs and GA can be self-administered, subcutaneously or intramuscularly), except for natalizumab (€589.78: weighted average tariff of ambulatory administration [38], 80% of cases, and day-hospital administration [39], 20% of cases). Annual monitoring costs were calculated assuming that the patients would comply with the main recommendations for RRMS. Clinical guidelines issued by the Emilia Romagna Region [40, 41] were used to estimate the cost of follow-up. Table 3 shows monitoring costs, by treatment. To estimate the cost of a relapse, data from Kobelt et al. [2] was used. The relapse management estimated cost (€4000) was adjusted for inflation to November 2015 (€4744). The economic impact of AEs was calculated assuming that mild-to-moderate events would be managed either by the general practitioner (GP) [42] or by the specialist [38], while severe events were managed in day-hospital or through standard hospitalization [39], depending on the event (expert opinion).

**Table 3:** Therapy, follow-up and administration costs [9, 43–50]

Treatment and posology	Ex-factory price (€) per pack^a^	Annual monitoring costs, Year 1 (€)	Annual monitoring costs, subsequent years (€)	Annual administration costs (€)	Source of ex-factory price
Natalizumab – Tysabri, 300 mg, Q4W	1800.00	1104.69	421.42	589.78	Gazette 292, 2006 [9] Gazette 139, 2014 [43]
IFN beta 1a - Rebif 44 mcg 44mcg, tiw	1027.75	1084.04	399.22	0.00	Gazette 196, 2009 [44] Gazette 274, 2011 [45]
IFN beta 1b – Betaferon, 250 mcg dieb. alt.	856.01	1084.04	399.22	0.00	Gazette 127, 2000 [46] Gazette 279, 2007 [47]
Glatiramer acetate – Copaxone, 20 mg, od	769.30	932.51	313.18	0.00	Gazette 106, 2005 [48]
IFN beta 1a – Avonex, 30 mcg, QW	790.17	1084.04	399.22	0.00	Gazette 11, 2004 [49] Gazette 272, 2011 [50]

*Tiw* three times a week, *dieb. alt* every other day, *od* once daily, *QW* every week, *Q4W* every four weeks, *IFN* interferon

^**a**^ It does not include temporary law reductions and any discount applied to public structures of Italian NHS

### Sensitivity analysis

Both univariate deterministic and probabilistic sensitivity analyses were conducted to identify the effects of input variability on the overall results of the analysis. The deterministic sensitivity analysis tested the effect of the following parameters: i) +/− 10% therapy costs; ii) +/− 10% direct disability costs; iii) +/− 10% relapse costs; iv) +/− 10% utility values; v) initiation of natalizumab at EDSS> = 3.0 in the SWI group; vi) initiation of natalizumab at EDSS> = 5.0 in the SWI group. For the probabilistic analysis, the following distributions were used: lognormal for clinical variables (relapse rates, adverse event rates, mortality risk increase due to MS, utilities and disutilities); beta for EDSS transition probabilities (in the switching and escalation groups); gamma for costs. A 10% standard error of the mean value of each variable was used to run probabilistic sensitivity analysis.

## Results

Table 4 shows the results of the analysis. Total lifetime social costs amounted to €699,676 and €718,604 in the ESC and SWI groups, respectively. In both groups, treatment costs and indirect costs were the most relevant drivers of expenditure, absorbing 85% of costs in the ESC group and 82% in the SWI group. The higher treatment and administration costs, monitoring costs and AE costs in the ESC group were offset by savings for the reduction of relapse burden and the delaying of disability progression, both reducing direct and indirect costs. The economic burden of AEs was negligible. Early adoption of escalation to natalizumab was more effective than switching among immunomodulators (IFNs and GA), leading to an increase of the discounted quality-adjusted survival (+ 1.52 QALYs; 11.19 in the ESC group, vs. 9.67 in the SWI group). The cost-effectiveness analysis showed that ESC dominated SWI in the societal perspective, being associated with lower costs and longer quality-adjusted survival. Early escalation of RRMS patients determined prolonged survival at a lower level of disability.

**Table 4:** Results of the cost-effectiveness analysis

Type of costs	ESC (B), €	SWI (A), €	Absolute difference (B-A), €	Relative difference (B/A), %
Treatment costs (€) + Administration costs (€)	327,938	236,288	91,650	38.79%
Monitoring costs (€)	7335	6278	1057	16.84%
Relapse costs (€)	41,938	57,350	−15,412	−26.87%
Adverse event costs (€)	1472	913	559	61.19%
EDSS direct costs (€)	42,881	57,884	−15,003	−25.92%
EDSS indirect costs (€)	278,113	359,891	−81,778	−22.72%
Total direct costs (€)	421,563	358,713	62,850	17.52%
Total social costs (€)	699,676	718,604	−18,928	−2.63%
Outcomes	ESC (B)	SWI (A)	Absolute difference (B-A)	Relative difference (B/A), %
Total QALYs	11.19	9.67	1.52	15.73%
Total LYs	20.10	19.67	0.43	2.20%
Incremental cost-effectiveness ratio (ICER)	Escalation (B) vs. Switching (A): Cost per outcome gained			
QALYs (social cost)	(ESC DOMINANT)			
LYs (social cost)	(ESC DOMINANT)			

*SWI* switching (group), *ESC* escalation (group), *EDSS* expanded disability status scale, *QALY* quality-adjusted life year, *LY* life year

**Fig. 2:**
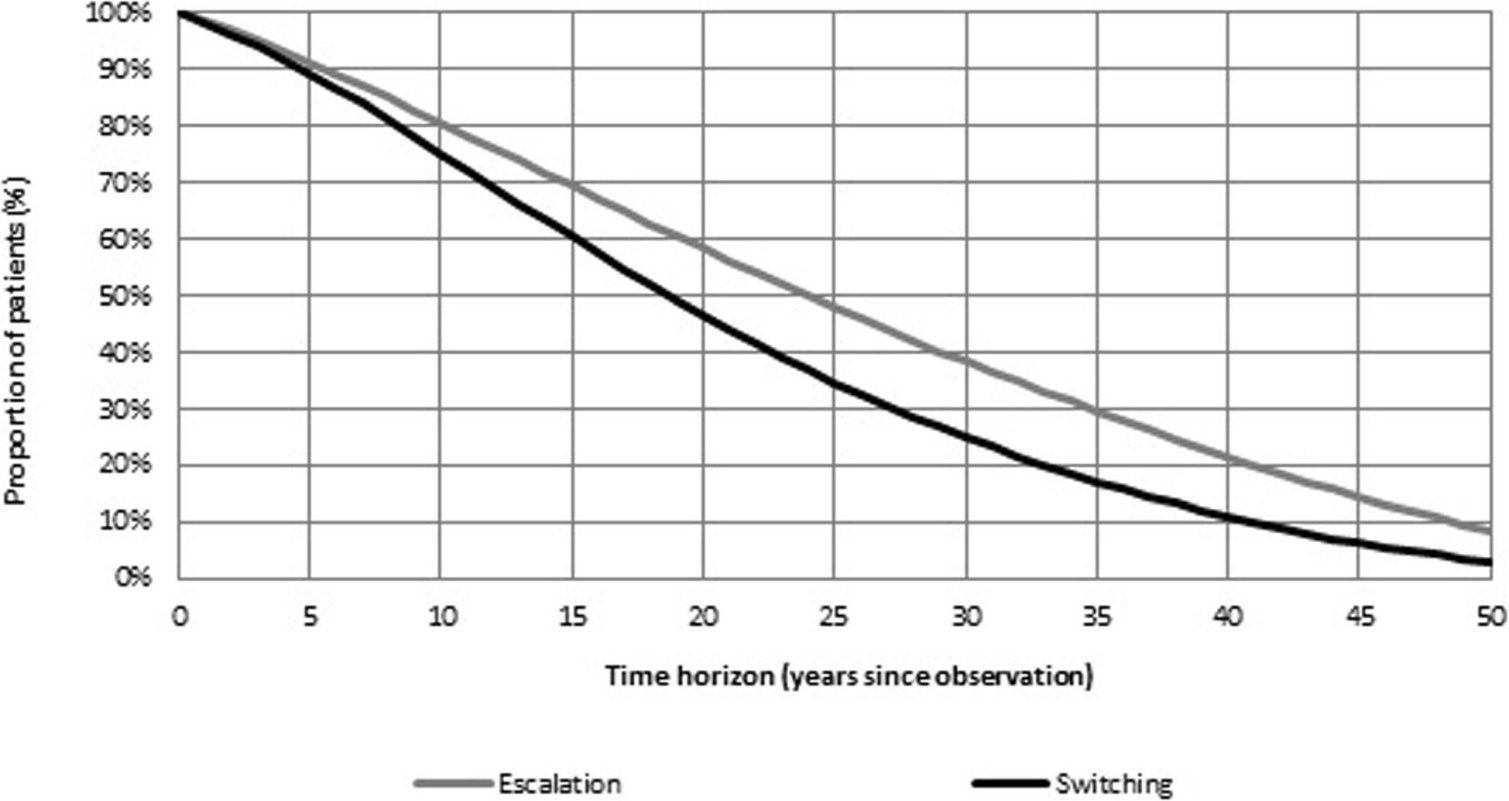
Proportion of patients with EDSS< 5, over time

**Fig. 3:**
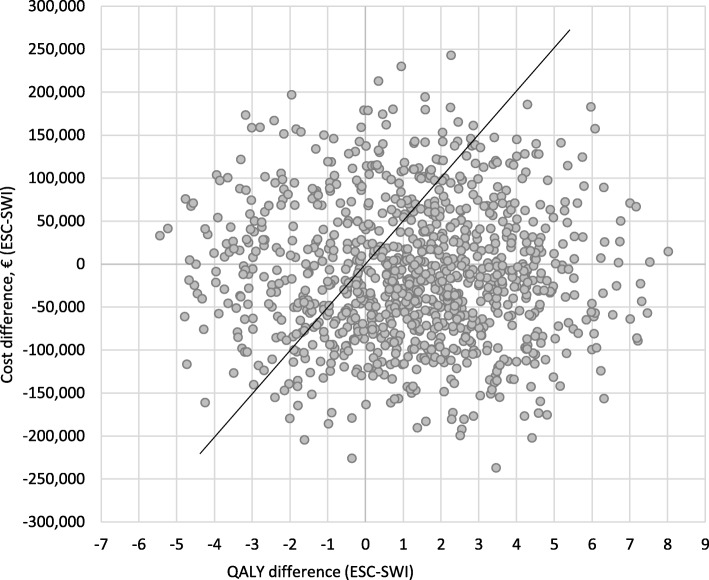
Results of the probabilistic sensitivity analysis. ESC = escalation strategy; SWI = switching strategy. Willingness-to-pay threshold at €50,000 per QALY gained

This result is shown in Fig. 2: after 15 years of observation, about 60% patients in the ESC group and 70% in the SWI group have an EDSS score greater than 5 (absolute reduction: − 10%). The clinical difference between the two groups reaches its maximum at around Year 30. Then the effect of mortality, similar in the two groups, progressively reduces the benefit. Similarly, the effect of relapse occurrence was reduced in the ESC group. The cumulative analysis conducted on relapses showed that, at the end of the observation, patients in the ESC group experienced fewer episodes than patients in the SWI group (8.84 vs. 12.09, respectively). For all tested scenarios, the results of the univariate sensitivity analysis confirmed early escalation to natalizumab being dominant over switching among IFNs and GA (Additional file 1: Table S7). Finally, probabilistic analysis (*N* = 1000 simulations) showed that ESC was cost-effective vs. SWI in 85.9% of cases, using a willingness to pay threshold of € 50,000 per QALY gained [51, 52]. In 54.4% of cases, the escalation strategy dominated the switching strategy (Fig. 3).

## Discussion

The broad experience achieved with natalizumab in pivotal clinical trials and in observational studies offers obust evidence of its effectiveness in RRMS patients (both naïve or who failed first-line DMT). Recently, the European Medicine Agency (EMA) approved the extension of natalizumab indication in patients with highly active RRMS despite a full and adequate course of treatment with at least one DMT, thus simplifying conditions for escalation [53]. Moreover, the recent review of the benefit-risk profile of natalizumab, conducted by EMA, led to the renewal of the marketing authorization with unlimited validity [54]. The present analysis provides evidence that escalation to natalizumab is a cost-effective option vs. switching among immunomodulators in RRMS patients who failed first-line therapy (IFNs, GA). To our knowledge, this is the first attempt of evaluating economic consequences of early escalation vs. switching in Italy. The clinical data used to conduct the present analysis were mainly derived from an observational analysis conducted in Italy. Each economic assessment is a dynamic process because: i) clinical efficacy data must be confirmed in real practice; ii) certain input data, such as prices of treatments, can change over time; iii) new technologies become available for patients. With this cost-utility analysis we were able to capture two relevant outcomes of the health technology assessment: costs and quality of life. Results of the analysis show that pharmacological expenditure is a relevant cost for healthcare services, but there are other non-negligible costs contributing to healthcare and social economic impact. In fact, although acquisition costs with natalizumab were substantially higher than IFNs and GA in our analysis, early escalation produced savings which entirely offset the investment with natalizumab, thus making escalation a dominant (i.e. less expensive and more effective) alternative vs. switching. Moreover, early escalation led to improved patient quality of life, prolonged survival with lower disability and slightly prolonged overall survival. Finally, these findings were confirmed in all tested scenarios (base-case, one-way sensitivity analysis, probabilistic analysis). In our base case analysis, we decided to adopt a societal perspective, as we aimed to capture the significant economic burden of multiple sclerosis, beyond costs sustained by our healthcare service. Indeed, in their Italian cost-of-illness assessment, Kobelt et al. [2] showed that direct healthcare costs of MS accounted for 28.6% of total per-patient costs, with total direct non-medical costs and productivity loss costs contributing for the remaining 42.3 and 29.1%, respectively.

Despite long-term modelling of costs and clinical consequences can be affected by some methodological limitations (iteration of clinical efficacy results, typically adopted in Markov models, provides a “proxy” of the real evolution of the disease) we believe this analysis is robust enough in terms of clinical efficacy model inputs (most of them were retrieved from published literature) and cost assumptions (retrieved from published literature and consistent with previous Italian publications on RRMS). Likely, the main limitation of this analysis concerns the use of data from a general RRMS population to estimate the natural history of the disease of a highly active RRMS population. Of course, the use of more specific data on highly active RRMS patients would have allowed a more accurate estimate of the clinical outcomes. Unfortunately, to our knowledge, such data are not available. However, we believe this bias disfavoured the option of escalating to natalizumab. Intuitively, the clinical benefits of a therapy reducing disability should be even more evident in a cohort of patients with highly active disease, characterized by faster disability progression. A second limitation of the study was that we did not use EDSS-dependant transition probabilities to model disease progression in the RRMS disease state. We adopted this approach as we did not have adequate sample size in the original study to calculate disability progression rates by EDSS state. Nevertheless, we believe that the benefit of using “real-data” on this specific target population would exceed the drawback of the lack of EDSS-dependant transition probabilities. Moreover, we tested the variability of this parameter in our sensitivity analyses, to get confirmation that the uncertainty associated with this variable would not determine a significant modification of the ICER estimates. Considering all these factors, we acknowledge that the present economic analysis does not represent a conclusive guidance for early treatment of natalizumab, which depends on several clinical factors. The findings of this analysis should be rather considered as a proof of the fact that the current economic evidence supports the escalation of natalizumab in the Italian setting, when this approach is considered appropriate from a clinical perspective. Furthermore, this economic analysis is adopting the Italian societal perspective. Therefore, caution should be taken around the validity of such conclusions in other countries.

## Conclusions

The results of this study showed that early escalation to natalizumab in RRMS patients who do not respond adequately to conventional immunomodulators (IFNs, GA) led to both clinical and economic benefits, compared to switching among immunomodulators (IFNs, GA). Overall, the escalation approach: i) improved quality of life, ii) prolonged survival with lower disability, and iii) slightly prolonged overall survival. Although natalizumab acquisition costs were substantially higher than IFNs and GA, early escalation produced savings. Taking together, our findings showed that early escalation to natalizumab was a dominant option for the treatment of RRMS patients who do not respond adequately to conventional immunomodulators (IFNs, GA).

## Additional file

Additional file 1: Description of data: inputs used in cost-effectiveness model. (DOCX 48 kb)

## Data Availability

The main data source of this analysis is the study Prosperini et al., Mult Scler 2012 (available at: 10.1177/1352458511417481).

## Abbreviations

AEs: Adverse events
ARR: Annualised relapse rate
DMT: Disease modify therapy
EDSS: Expanded Disability Status
EMA: European Medicine Agency
ESC: Patients receiving natalizumab
GA: Glatiramer acetate
ICER: Incremental cost effectiveness ratio
IFN: Interferon beta
ISTAT: Italian Statistics Institute
MS: Multiple Sclerosis
MTC: Mixed treatment comparison
NHS: National Healthcare System
NICE: National Institute of Health and Care Excellence
QALYs: Quality-adjusted survival
RR: Relapsing-remitting
RRMS: Relapsing-remitting multiple sclerosis
SPMS: Secondary progressive multiple sclerosis
SWI: Patients switching among different IFN formulations, or from IFN to GA, or vice versa
