# Supplementary material for: Cost-effectiveness analysis of escalating to natalizumab or switching among immunomodulators in relapsing-remitting multiple sclerosis in Italy
Source: BMC Health Serv Res. 2019 Jun 28;19:436. doi: 10.1186/s12913-019-4264-1 (PMC6599237; doi:10.1186/s12913-019-4264-1)
Supplement: Supplementary file 1 — Description of data: inputs used in cost-effectiveness model. (DOCX 48 kb) [file 12913_2019_4264_MOESM1_ESM.docx]

Table 1. List of the input data used in the cost-effectiveness model.

| **Input** | **ESC** | **SWI** | **Source** |
| --- | --- | --- | --- |
| Number of patients (n) | 1 | | Assumption |
| Time horizon (years) | 50 | | Assumption |
| Discount rate, outcomes (%) | 3.50% | | NICE, 2013 [21] |
| Discount rate, costs (%) | 3.50% | | NICE, 2013 [21] |
| Age of the cohort (years + standard deviation) | 35.3 + 8.3 | | Prosperini et al., 2012 [6] |
| Proportion of female pts (%) | 65.8% | | Prosperini et al.,2012 [6] |
| EDSS distribution at baseline (%) | EDSS 0: 0.0%  EDSS 1: 25.6%  EDSS 2: 37.2%  EDSS 3: 20.7%  EDSS 4: 10.2%  EDSS 5: 6.3%  EDSS >6: 0.0% | | Prosperini et al.,2012 [6] |
| Relapse rate in the RRMS group, if not treated, by EDSS (events per patient/year) | EDSS 0-1: 1.7534  EDSS 2: 1.6698  EDSS 3: 1.7966  EDSS 4: 1.3793  EDSS 5-7: 1.5556  EDSS >8: 0.1555* | | EDSS 0-7: Prosperini et al., 2012 [6];  EDSS>8: Hutchinson et al., 2013 [22]* |
| Relapse rate in the SPMS group, if not treated, by EDSS (events per patient/year) | EDSS 0-1: 0.0000  EDSS 2: 0.3147  EDSS 3: 0.6020  EDSS 4: 0.5146  EDSS 5: 0.1604  EDSS 6: 0.1387  EDSS >7: 0.1041 | | Elaboration from Hutchinson et al., 2013 [22] |
| Mortality rate, by age | Mortality (by age) tables | | ISTAT, 2014 [32] |
| Mortality risk increase due to RRMS or SPMS, by EDSS | EDSS 0: 1.000  EDSS 1: 1.3000  EDSS 2: 1.6000  EDSS 3: 1.6800  EDSS 4: 1.7600  EDSS 5: 1.8400  EDSS 6: 2.7067  EDSS 7: 3.5733  EDSS 8: 4.4400  EDSS >9: 5.3067 | | Pokorski et al., 1997 [33] |
| Distribution of patients by treatment, in the SWI group (%) | Glatiramer acetate: 32.9%  IFN beta 1a IM: 0.0%  IFN beta 1a SC22: 0.0%  IFN beta 1a SC44: 58.4%  IFN beta 1b: 8.7% | | Prosperini et al., 2012 [6] |
| EDSS score at which natalizumab is interrupted | EDSS 7 | | Assumption |
| SWI group - EDSS score at which escalation to Tysabri occurs | N/A | EDSS 4 | Assumption |
| Treatment effect on annualised relapse rate, relative to placebo | 0.3024 | 0.6574 | Elaboration from Prosperini et al.,2012 [6] |
| RRMS transition probabilities  (EDSS level) | -1 pt: 0.0236  0 pt: 0.8727  +1 pt: 0.0849  +2 pt: 0.0189  +3 pt: 0.0000 | -1 pt: 0.0000  0 pt: 0.8199  +1 pt: 0.1553  +2 pt: 0.0217  +3 pt: 0.0031 | Elaboration from Prosperini et al., 2012 [6] |
| EDSS transition matrix -  SPMS, natural history | See Additional file 1 Table 2 | | Elaboration from: Weinshenker et al., 1989 [28], Cottrell et al., 1999 [29] and Kremenchutzky et al., 2006 [30] |
| EDSS transition matrix - transition from RRMS to SPMS | See Additional file 1 Table 3 | |  |

Table 2. List of the utility and safety data used in the cost-effectiveness model.

| **Input** | **ESC** | **SWI** | **Source** |
| --- | --- | --- | --- |
| Utilities, by EDSS status, RRMS from | EDSS 0: 0.8752  EDSS 1: 0.8342  EDSS 2: 0.7802  EDSS 3: 0.6946  EDSS 4: 0.6253  EDSS 5: 0.5442  EDSS 6: 0.4555  EDSS 7: 0.3437  EDSS 8: 0.0023  EDSS >9: -0.1701 | | Elaboration from: Gold et al., 2012 [34]; Fox et al.,2012 [35] and UK Multiple Sclerosis Survey, 2007 [36] |
| Utilities, by EDSS status, SPMS form | EDSS 0: 0.8660  EDSS 1: 0.8250  EDSS 2: 0.7710  EDSS 3: 0.6855  EDSS 4: 0.6161  EDSS 5: 0.5350  EDSS 6: 0.4463  EDSS 7: 0.3346  EDSS 8: -0.0068  EDSS >9: -0.1793 | |  |
| Disutility, per relapse | -0.0437 | | UK Multiple Sclerosis Survey, 2007 [36] |
| Disutility, per PML | -0.300 | | Expert opinion |
| Non-serious AE RATE - Abdominal pain | 5.66% | 0.20% | Elaboration from: Hutchinson et al., 2013 [22]; Furneri et al.,2016 [24] |
| Non-serious AE RATE - Abdominal pain upper | 0.00% | 0.20% |  |
| Non-serious AE RATE - ALT increased | 0.00% | 8.44% |  |
| Non-serious AE RATE - Arthralgia | 9.99% | 3.26% |  |
| Non-serious AE RATE - Atrioventricular conduction block | 0.00% | 0.10% |  |
| Non-serious AE RATE - Back pain | 0.00% | 6.03% |  |
| Non-serious AE RATE - Bradycardia | 0.00% | 0.10% |  |
| Non-serious AE RATE - Chest pain | 2.50% | 0.57% |  |
| Non-serious AE RATE - | 0.00% | 0.48% |  |
| Non-serious AE RATE - Depression | 9.99% | 8.97% |  |
| Non-serious AE RATE - Diarrhea | 0.00% | 9.30% |  |
| Non-serious AE RATE - Fatigue | 14.53% | 14.24% |  |
| Non-serious AE RATE - Flu-like symptoms | 0.00% | 28.51% |  |
| Non-serious AE RATE - Flushing | 0.00% | 0.54% |  |
| Non-serious AE RATE - Gastroenteritis | 5.58% | 0.25% |  |
| Non-serious AE RATE - Headache | 21.23% | 24.53% |  |
| Non-serious AE RATE - Influenza | 8.85% | 5.19% |  |
| Non-serious AE RATE - Leukopenia | 0.00% | 7.36% |  |
| Non-serious AE RATE - Lower respiratory tract infections | 8.93% | 0.05% |  |
| Non-serious AE RATE - Nausea | 0.00% | 6.67% |  |
| Non-serious AE RATE - Pain in extremity | 0.00% | 22.14% |  |
| Non-serious AE RATE - Pruritus | 2.01% | 2.12% |  |
| Non-serious AE RATE - Rash | 5.66% | 6.44% |  |
| Non-serious AE RATE - Urinary tract infection | 10.52% | 6.71% |  |
| Serious AE RATE - Depression | 0.00% | 0.11% |  |
| Serious AE RATE - Gastroenteritis | 0.08% | 0.00% |  |
| Serious AE RATE - Influenza | 0.09% | 0.00% |  |
| Non-serious AE DISUTILITY - Abdominal pain | 0.0000 | | Elaboration from: Hutchinson et al., 2013 [22]; Furneri et al.,2016 [24] |
| Non-serious AE DISUTILITY - Abdominal pain upper | 0.0000 | |  |
| Non-serious AE DISUTILITY - ALT increased | 0.0000 | |  |
| Non-serious AE DISUTILITY - Arthralgia | 0.0000 | |  |
| Non-serious AE DISUTILITY - Atrioventricular conduction block | 0.0008 | |  |
| Non-serious AE DISUTILITY - Back pain | 0.0072 | |  |
| Non-serious AE DISUTILITY - Bradycardia | 0.0000 | |  |
| Non-serious AE DISUTILITY - Chest pain | 0.0048 | |  |
| Non-serious AE DISUTILITY - | 0.0000 | |  |
| Non-serious AE DISUTILITY - Depression | 0.0338 | |  |
| Non-serious AE DISUTILITY - Diarrhea | 0.0000 | |  |
| Non-serious AE DISUTILITY - Fatigue | 0.0000 | |  |
| Non-serious AE DISUTILITY - Flu-like symptoms | 0.0223 | |  |
| Non-serious AE DISUTILITY - Flushing | 0.0000 | |  |
| Non-serious AE DISUTILITY - Gastroenteritis | 0.0017 | |  |
| Non-serious AE DISUTILITY - Headache | 0.0040 | |  |
| Non-serious AE DISUTILITY - Influenza | 0.0181 | |  |
| Non-serious AE DISUTILITY - Leukopenia | 0.0000 | |  |
| Non-serious AE DISUTILITY - Lower respiratory tract infections | 0.0016 | |  |
| Non-serious AE DISUTILITY - Nausea | 0.0000 | |  |
| Non-serious AE DISUTILITY - Pain in extremity | 0.0048 | |  |
| Non-serious AE DISUTILITY - Pruritus | 0.0000 | |  |
| Non-serious AE DISUTILITY - Rash | 0.0000 | |  |
| Non-serious AE DISUTILITY - Urinary tract infection | 0.0014 | |  |
| Serious AE DISUTILITY - Abdominal pain | 0.0000 | |  |
| Serious AE DISUTILITY - Abdominal pain upper | 0.0000 | |  |
| Serious AE DISUTILITY - ALT increased | 0.0000 | |  |
| Serious AE DISUTILITY - Arthralgia | 0.0168 | |  |
| Serious AE DISUTILITY - Atrioventricular conduction block | 0.0008 | |  |
| Serious AE DISUTILITY - Back pain | 0.0336 | |  |
| Serious AE DISUTILITY - Bradycardia | 0.0000 | |  |
| Serious AE DISUTILITY - Chest pain | 0.0192 | |  |
| Serious AE DISUTILITY - | 0.0000 | |  |
| Serious AE DISUTILITY - Depression | 0.5604 | |  |
| Serious AE DISUTILITY - Diarrhea | 0.0000 | |  |
| Serious AE DISUTILITY - Fatigue | 0.0000 | |  |
| Serious AE DISUTILITY - Flu-like symptoms | 0.0446 | |  |
| Serious AE DISUTILITY - Flushing | 0.0000 | |  |
| Serious AE DISUTILITY - Gastroenteritis | 0.0017 | |  |
| Serious AE DISUTILITY - Headache | 0.0331 | |  |
| Serious AE DISUTILITY - Influenza | 0.0450 | |  |
| Serious AE DISUTILITY - Leukopenia | 0.0000 | |  |
| Serious AE DISUTILITY - Lower respiratory tract infections | 0.0016 | |  |
| Serious AE DISUTILITY - Nausea | 0.0000 | |  |
| Serious AE DISUTILITY - Pain in extremity | 0.0192 | |  |
| Serious AE DISUTILITY - Pruritus | 0.0000 | |  |
| Serious AE DISUTILITY - Rash | 0.0000 | |  |
| Serious AE DISUTILITY - Urinary tract infection | 0.0014 | |  |
| Non-serious AE COST (€) - Abdominal pain | 20.7 | | Elaboration from: Ministry of Health inpatient and outpatient tariffs, 2013 [38-39], Garattini et al., 2003 [41] and ISTAT inflation rates [37]. |
| Non-serious AE COST (€) - Abdominal pain upper | 20.7 | |  |
| Non-serious AE COST (€) - ALT increased | 1.0 | |  |
| Non-serious AE COST (€) - Arthralgia | 15.7 | |  |
| Non-serious AE COST (€) - Atrioventricular conduction block | 191.8 | |  |
| Non-serious AE COST (€) - Back pain | 15.7 | |  |
| Non-serious AE COST (€) - Bradycardia | 191.8 | |  |
| Non-serious AE COST (€) - Chest pain | 20.7 | |  |
| Non-serious AE COST (€) - | 15.7 | |  |
| Non-serious AE COST (€) - Depression | 87.6 | |  |
| Non-serious AE COST (€) - Diarrhea | 0.0 | |  |
| Non-serious AE COST (€) - Fatigue | 0.0 | |  |
| Non-serious AE COST (€) - Flu-like symptoms | 0.0 | |  |
| Non-serious AE COST (€) - Flushing | 20.7 | |  |
| Non-serious AE COST (€) - Gastroenteritis | 183.8 | |  |
| Non-serious AE COST (€) - Headache | 0.0 | |  |
| Non-serious AE COST (€) - Influenza | 0.0 | |  |
| Non-serious AE COST (€) - Leukopenia | 3.9 | |  |
| Non-serious AE COST (€) - Lower respiratory tract infections | 215.4 | |  |
| Non-serious AE COST (€) - Nausea | 0.0 | |  |
| Non-serious AE COST (€) - Pain in extremity | 20.7 | |  |
| Non-serious AE COST (€) - Pruritus | 0.0 | |  |
| Non-serious AE COST (€) - Rash | 20.7 | |  |
| Non-serious AE COST (€) - Urinary tract infection | 20.7 | |  |
| Serious AE COST (€) - Abdominal pain | 20.7 | |  |
| Serious AE COST (€) - Abdominal pain upper | 20.7 | |  |
| Serious AE COST (€) - ALT increased | 21.7 | |  |
| Serious AE COST (€) - Arthralgia | 20.7 | |  |
| Serious AE COST (€) - Atrioventricular conduction block | 1,542.1 | |  |
| Serious AE COST (€) - Back pain | 20.7 | |  |
| Serious AE COST (€) - Bradycardia | 1,542.1 | |  |
| Serious AE COST (€) - Chest pain | 1,398.4 | |  |
| Serious AE COST (€) - | 20.7 | |  |
| Serious AE COST (€) - Depression | 1,198.3 | |  |
| Serious AE COST (€) - Diarrhea | 20.7 | |  |
| Serious AE COST (€) - Fatigue | 20.7 | |  |
| Serious AE COST (€) - Flu-like symptoms | 20.7 | |  |
| Serious AE COST (€) - Flushing | 20.7 | |  |
| Serious AE COST (€) - Gastroenteritis | 1,396.5 | |  |
| Serious AE COST (€) - Headache | 194.9 | |  |
| Serious AE COST (€) - Influenza | 215.6 | |  |
| Serious AE COST (€) - Leukopenia | 24.6 | |  |
| Serious AE COST (€) - Lower respiratory tract infections | 5,438.5 | |  |
| Serious AE COST (€) - Nausea | 20.7 | |  |
| Serious AE COST (€) - Pain in extremity | 31.0 | |  |
| Serious AE COST (€) - Pruritus | 20.7 | |  |
| Serious AE COST (€) - Rash | 217.9 | |  |
| Serious AE COST (€) - Urinary tract infection | 2,268.6 | |  |

Table 3. List of PML-related data used in the cost-effectiveness model.

| **Input** | **ESC** | **SWI** | **Source** |
| --- | --- | --- | --- |
| Distribution of pts, by JCV status (%) | JCV + : 45%  JCV - : 55% | N/A | Elaboration from Biogen post-marketing surveillance, 2014 [31] |
| Patients with immunosuppressant (IS) treatment history (%) | 2.0% | N/A |  |
| Annual PML risk 0-24 months, JCV - | 0.0100% | N/A |  |
| Annual risk 0-24 JCV +, no prior IS use | 0.0700% | N/A |  |
| Annual risk 0-24 JCV +, prior IS use | 0.1800% | N/A |  |
| Annual risk >24 JCV - | 0.0100% | N/A |  |
| Annual risk >24 JCV +, no prior IS use | 0.5700% | N/A |  |
| Annual risk >24 JCV +, prior IS use | 1.1200% | N/A |  |
| PML fatality | 22.0% | N/A |  |
| Cost of fatal PML (€) | 50,665 | N/A | Elaboration from: Ministry of Health inpatient tariffs, 2013  [38-39] |
| Cost of non-fatal PML (€) | 2,850 | N/A |  |
| Mortality rate, by age | Mortality (by age) tables | | ISTAT, 2014 [32] |

Table 4. List of cost data used in the cost-effectiveness model.

| **Input** | **ESC** | **SWI** | **Source** |
| --- | --- | --- | --- |
| Annual treatment cost per year (€) | 20,168 | 10,509 | Elaboration from Italian Medicines Agency [9, 43-50] |
| Annual administration costs (€) | 589.78 | 0 | Elaboration from: Ministry of Health inpatient tariffs, 2013  [38-39] |
| Annual monitoring costs (€) | 421.42 | 370.90 | Elaboration from: Ministry of Health outpatient tariffs, 2013 [38], Emilia-Romagna Region recommendations on monitoring, 2011 [40], and Friuli Venezia Giulia Region outpatient tariffs, 2015 [41] |
| Relapse unit cost (€) | 4,744 | | Elaboration from Kobelt et al., 2006 [2] and ISTAT inflation rates [37] |
| Annual EDSS-related direct costs (€) RRMS form | EDSS 0-2: 201  EDSS 3-6: 636  EDSS >7: 5,708 | | Elaboration from Karampampa et al., 2012 [1] and ISTAT inflation rates [37] |
| Annual EDSS-related indirect costs (€) RRMS form | EDSS 0-2: 1,143  EDSS 3-6: 11,847  EDSS >7: 28,411 | |  |
| Annual EDSS-related direct costs (€) SPMS form | EDSS 0-2: 5,331  EDSS 3-6: 18,894  EDSS >7: 9,589 | |  |
| Annual EDSS-related indirect costs (€) SPMS form | EDSS 0-2: 4,096  EDSS 3-6: 31,559  EDSS >7: 64,948 | |  |

SWI: switching (group). ESC: escalation (group). EDSS: expanded disability status scale. RRMS: relapsing-remitting multiple sclerosis. SPMS: secondary progressive multiple sclerosis. NICE: National Institute for Health and Care Excellence. IFN: interferon. ISTAT: Italian National Institute of Statistics. PML: progressive multifocal leukoencephalopathy. AE: adverse event. JCV: John Cunningham virus. IS: immunosuppressant.

Table 5. EDSS transition matrix, SPMS natural history
[Sources reported in Additional file 1 Table 1].

| From EDSS / to EDSS | 0 | 1 | 2 | 3 | 4 | 5 | 6 | 7 | 8 | 9 | 10 |
| --- | --- | --- | --- | --- | --- | --- | --- | --- | --- | --- | --- |
| 0 | 1.0000 | 0.0000 | 0.0000 | 0.0000 | 0.0000 | 0.0000 | 0.0000 | 0.0000 | 0.0000 | 0.0000 | 0.0000 |
| 1 | 0.0000 | 0.7692 | 0.1538 | 0.0769 | 0.0000 | 0.0000 | 0.0000 | 0.0000 | 0.0000 | 0.0000 | 0.0000 |
| 2 | 0.0000 | 0.0000 | 0.6357 | 0.2713 | 0.0620 | 0.0233 | 0.0078 | 0.0000 | 0.0000 | 0.0000 | 0.0000 |
| 3 | 0.0000 | 0.0000 | 0.0000 | 0.6291 | 0.2527 | 0.0769 | 0.0330 | 0.0027 | 0.0055 | 0.0000 | 0.0000 |
| 4 | 0.0000 | 0.0000 | 0.0000 | 0.0000 | 0.4854 | 0.3504 | 0.1387 | 0.0073 | 0.0182 | 0.0000 | 0.0000 |
| 5 | 0.0000 | 0.0000 | 0.0000 | 0.0000 | 0.0000 | 0.6325 | 0.3173 | 0.0221 | 0.0261 | 0.0020 | 0.0000 |
| 6 | 0.0000 | 0.0000 | 0.0000 | 0.0000 | 0.0000 | 0.0000 | 0.7631 | 0.1903 | 0.0446 | 0.0020 | 0.0000 |
| 7 | 0.0000 | 0.0000 | 0.0000 | 0.0000 | 0.0000 | 0.0000 | 0.0000 | 0.8046 | 0.1891 | 0.0062 | 0.0000 |
| 8 | 0.0000 | 0.0000 | 0.0000 | 0.0000 | 0.0000 | 0.0000 | 0.0000 | 0.0000 | 0.9258 | 0.0742 | 0.0000 |
| 9 | 0.0000 | 0.0000 | 0.0000 | 0.0000 | 0.0000 | 0.0000 | 0.0000 | 0.0000 | 0.0000 | 1.0000 | 0.0000 |
| 10 | 0.0000 | 0.0000 | 0.0000 | 0.0000 | 0.0000 | 0.0000 | 0.0000 | 0.0000 | 0.0000 | 0.0000 | 1.0000 |

EDSS: expanded disability status scale. SPMS: secondary progressive multiple sclerosis.

Table 6. EDSS transition matrix, transition from RRMS to SPMS
[Sources reported in Additional file 1 Table 1].

| From EDSS / to EDSS | Probability |
| --- | --- |
| 0 🡪 1 | 0.0000 |
| 1 🡪 2 | 0.0030 |
| 2 🡪 3 | 0.0321 |
| 3 🡪 4 | 0.1166 |
| 4 🡪 5 | 0.2104 |
| 5 🡪 6 | 0.2987 |
| 6 🡪 7 | 0.2372 |
| 7 🡪 8 | 0.2535 |
| 8 🡪 9 | 0.1525 |
| 9 🡪 10 | 1.0000 |

EDSS: expanded disability status scale. RRMS: relapsing-remitting multiple sclerosis.
SPMS: secondary progressive multiple sclerosis.

Table 7. Results of the one-way sensitivity analysis.

| Analysis | Cost difference (ESC-SWI), € | QALY difference (ESC-SWI) | ICER |
| --- | --- | --- | --- |
| Base-case | -18,928 | 1.52 | ESC dominates |
| +10% treatment costs | -10,205 | 1.52 | ESC dominates |
| -10% treatment costs | -27,651 | 1.52 | ESC dominates |
| +10% disability direct costs | -20,428 | 1.52 | ESC dominates |
| -10% disability direct costs | -17,428 | 1.52 | ESC dominates |
| +10% disability indirect costs | -27,106 | 1.52 | ESC dominates |
| -10% disability indirect costs | -10,705 | 1.52 | ESC dominates |
| +10% relapse costs | -20,469 | 1.52 | ESC dominates |
| -10% relapse costs | -17,387 | 1.52 | ESC dominates |
| +10% utility values | -18,928 | 1.66 | ESC dominates |
| -10% utility values | -18,928 | 1.38 | ESC dominates |
| Initiation of natalizumab  at EDSS>=3.0 in the SWI group | -14,333 | 0.86 | ESC dominates |
| Initiation of natalizumab  at EDSS>=5.0 in the SWI group | -18,572 | 2.13 | ESC dominates |

SWI: switching (group). ESC: escalation (group). EDSS: expanded disability status scale. QALY: quality adjusted life years.
